# Clinical experiences in low-resource settings: Experience from Croatia

**DOI:** 10.1192/j.eurpsy.2021.152

**Published:** 2021-08-13

**Authors:** S. Medved, M. Rojnic-Kuzman

**Affiliations:** 1 Department Of Psychiatry And Psychological Medicine, University Hospital Centre Zagreb, Zagreb, Croatia; 2 Department Of Psychiatry And Psychological Medicine, Zagreb School of Medicine and University Hospital Centre Zagreb, Zagreb, Croatia

**Keywords:** Mental Health Services, pandemic, flexible assertive community treatment teams

## Abstract

Croatian healthcare faced dramatic changes as a response to the ongoing pandemic, which further pressured financially loaded system. Besides, the capital of Croatia was hit by an earthquake that caused material damage and organizational difficulties. Contrary to the World Health Organization’s recommendations that mental health services are essential services to be maintained during the pandemic, there was severe disruption of utilization and accessibility to mental health services during the pandemic. In the beginning, the only services maintained were in-hospital and emergency services, while daily hospitals and ambulatory visits were disrupted. Psychiatric resources were not formally implemented in the treatment and management of COVID-19 and majority of mental health workers remained within the boundaries of psychiatric services. In the following period, some of the services reopened, with reorientation to online provision. However, the interference of services gradually occurred as the second wave started, with some psychiatric departments being repurposed for non-psychiatric use. Psychiatric care was organized in some of the COVID-19 departments as liaison service. Some of the psychiatric services offered consultation and prevention of burn-out for frontline personnel. Lastly, as the University hospital centre Zagreb was implementing the flexible assertive community treatment teams for persons with severe mental illness through the Large-scale implementation of community based mental health care for people with severe and enduring mental ill health in Europe (RECOVER-E) project in the period from 2018-2022, we investigated the effect of this service on the health outcomes during the pandemic and found it superior compared with the standard treatment.

**Disclosure:**

No significant relationships.

